# Combining Short-Term Interval Training with Caloric Restriction Improves ß-Cell Function in Obese Adults

**DOI:** 10.3390/nu10060717

**Published:** 2018-06-03

**Authors:** Monique E. Francois, Nicole M. Gilbertson, Natalie Z. M. Eichner, Emily M. Heiston, Chiara Fabris, Marc Breton, J. Hunter Mehaffey, Taryn Hassinger, Peter T. Hallowell, Steven K. Malin

**Affiliations:** 1 Department of Kinesiology, University of Virginia, Charlottesville, VA 22903, USA; mf4rq@virginia.edu (M.E.F.); nmg4xk@virginia.edu (N.M.G.); nze8bz@virginia.edu (N.Z.M.E.); emh5bh@virginia.edu (E.M.H.); 2 Center for Diabetes Technology, School of Medicine, University of Virginia, Charlottesville, VA 22903, USA; cf9qe@virginia.edu (C.F.); mb6nt@virginia.edu (M.B.); 3 Department of Surgery, School of Medicine, University of Virginia, Charlottesville, VA 22903, USA; jhm9t@hscmail.mcc.virginia.edu (J.H.M.); teh3rz@virginia.edu (T.H.); PTH2F@hscmail.mcc.virginia.edu (P.T.H.); 4 Division of Endocrinology and Metabolism, University of Virginia, Charlottesville, VA 22903, USA; 5 Robert M. Berne Cardiovascular Research Center, University of Virginia, Charlottesville, VA, 22903, USA

**Keywords:** caloric restriction, diabetes, obesity, exercise, glucose control

## Abstract

Although low-calorie diets (LCD) improve glucose regulation, it is unclear if interval exercise (INT) is additive. We examined the impact of an LCD versus LCD + INT training on ß-cell function in relation to glucose tolerance in obese adults. Twenty-six adults (Age: 46 ± 12 year; BMI 38 ± 6 kg/m^2^) were randomized to 2-week of LCD (~1200 kcal/day) or energy-matched LCD + INT (60 min/day alternating 3 min at 90 and 50% HRpeak). A 2 h 75 g oral glucose tolerance test (OGTT) was performed. Insulin secretion rates (ISR) were determined by deconvolution modeling to assess glucose-stimulated insulin secretion ([GSIS: ISR/glucose total area under the curve (tAUC)]) and ß-cell function (Disposition Index [DI: GSIS/IR]) relative to skeletal muscle (Matsuda Index), hepatic (HOMA-IR) and adipose (Adipose-IR_fasting_) insulin resistance (IR). LCD + INT, but not LCD alone, reduced glucose and total-phase ISR tAUC (Interactions: *p* = 0.04 and *p* = 0.05, respectively). Both interventions improved skeletal muscle IR by 16% (*p* = 0.04) and skeletal muscle and hepatic DI (Time: *p* < 0.05). Improved skeletal muscle DI was associated with lower glucose tAUC (*r* = −0.57, *p* < 0.01). Thus, LCD + INT improved glucose tolerance more than LCD in obese adults, and these findings relate to ß-cell function. These data support LCD + INT for preserving pancreatic function for type 2 diabetes prevention.

## 1. Introduction

Obesity affects about 13% of adults worldwide and increases the risk for type 2 diabetes (T2D) [1,2]. While the relation between excess body fat and T2D is complex, the development of T2D is mainly characterized by insulin resistance that contributes to hyperinsulinemia and pancreatic ß-cell dysfunction [3,4]. Over time, it is the loss of ß-cell function that leads to impaired glucose tolerance and T2D [3,5]. The oral disposition index (DI) is a measure of pancreatic insulin secretion [6,7] and is a stronger predictor of future T2D risk than insulin sensitivity alone [8]. Therefore, interventions that improve pancreatic ß-cell dysfunction are essential to the prevention of T2D [9]. 

Caloric restriction is an established treatment for reducing obesity and lowering T2D risk. Adding exercise to caloric restriction interventions may be synergistic as they both independently reduce insulin resistance [10]. Caloric restriction has strong effects on reducing endogenous glucose-production and hepatic insulin resistance [11,12], whereas exercise has well-established benefits for skeletal muscle insulin-stimulated glucose uptake [13,14]. A recent long-term study that combined caloric restriction and exercise to elicit weight losses of 6−8% demonstrated a two-fold greater improvement in insulin sensitivity compared to the same weight loss achieved by diet or exercise alone, although there was no added benefit to ß-cell function [10]. However, prior work by some [15,16], but not all [14] suggests that exercise volume is important for pancreatic function in people with prediabetes, and recent work has highlighted that for a given exercise duration, high-intensity interval training provides added benefits for cardiometabolic health compared to moderate intensity exercise in T2D [17,18]. Interestingly, interval exercise training improves ß-cell function relative to changes in skeletal muscle insulin resistance in those with obesity and T2D [13,19]. Our group has previously demonstrated that an acute bout of high intensity exercise differentially impacts skeletal muscle versus liver and adipose indices of ß-cell function to maintain glucose homeostasis in the immediate post-exercise period when compared with moderate exercise in adults with prediabetes [20]. To date, it remains unknown if high-intensity interval exercise adds to the benefits of caloric restriction on pancreatic ß-cell function prior to clinically meaningful weight loss of approximately 7% in obese adults without overt hyperglycemia [21]. This is relevant as weight loss is often advocated to reduce T2D development by 58% [22] thereby confounding the ability to understand the acute preventative effects of lifestyle intervention. Therefore, we tested the hypothesis that combining interval exercise with a low-calorie diet would enhance ß-cell function compared to a matched caloric restriction diet alone in adults with obesity. Secondly, given the known effects of caloric restriction on the gut and the impact of incretin hormones on insulin secretion [23], we hypothesized that changes in GSIS adjusted for skeletal muscle, liver and/or adipose insulin resistance would correlate with improved GLP-1 and glucose tolerance following the interventions.

## 2. Materials and Methods

### 2.1. Participants

Twenty-six participants (Age: 46 ± 12 year, BMI: 38 ± 6 kg/m^2^, Table 1) were randomized to either 2-weeks of a low-calorie diet (LCD) or an energy matched LCD with interval exercise training (LCD + INT). Participants volunteered to participate in this study, and were recruited from advertisements in the Charlottesville, Virginia community. All participants underwent health screenings that included a resting and exercise stress test with 12-lead electrocardiogram (EKG), medical history, which indicated they were weight stable (<2 kg over last 3 m) and physical examination, as well as blood chemistry analysis. Individuals were excluded if pregnant or had known cardiovascular disease, T2D, cancer, contraindications to exercise (e.g., musculoskeletal injuries), and/or taking medications (e.g., metformin, acarbose, GLP-1 agonists, etc.) known to affect glucose homeostasis. A similar number of females were pre- (*n* = 6 LCD, *n* = 5 LCD + INT) and post-menopausal (*n* = 5 LCD, *n* = 5 LCD + INT) in each treatment group. All participants gave also their informed consent for inclusion before they participated in the study. The study was conducted in accordance with the Declaration of Helsinki, and the protocol was approved by the University of Virginia Ethics Committee (IRB-HSR # 18316).

### 2.2. Low-Calorie Diet (LCD)

Participants were prescribed an LCD (1000−1200 kcal/day) based on 2-week pre-operative diets recommended to obese adults undergoing bariatric surgery [24]. We elected this dietary intervention to develop pilot data for adults potentially undergoing bariatric surgery. To achieve this, participants were provided a meal replacement shake for breakfast and lunch (Ensure^®^ Abbott Laboratories, Lake Bluff, IL, USA, 8 fl. oz; providing 160 kcal, 16 g protein, 2 g fat, 19 g carbohydrate). Participants were then instructed on a sensible dinner option that did not exceed 600 kcal (e.g., lean protein with vegetables or salad). Additionally, two 100 kcal snack options were provided to account for the remaining caloric deficit. Detailed instructions for preparing, and recording food and beverages were given to participants before the intervention. Empty shake containers were returned/counted and 13 day food records were completed throughout the intervention and averaged to assess compliance (Table 1). Caloric deficit was calculated as the average of 3 day food logs pre-intervention minus the 13 day average of food intake. This approach does not account for the 350 kcal post-exercise energy intake.

### 2.3. Exercise Training

Participants randomized to LCD + INT performed 12 sessions of interval exercise (INT) over a 13-d period, with one rest day at the half-way point. Each exercise session was supervised and alternated 3 min periods of cycling at 50% and 90% of heart rate peak (HRpeak); ten repetitions of 50% and 90% were completed per 60 min session. The duration progressed from 30 min on day 1, to 45 min on day 2, and 60 min thereafter. After each exercise session, a mixed-meal shake (Ensure^®^ Abbott Laboratories, Lake Forest, IL, USA, 8 fl. oz; providing 350 kcal, 13 g protein, 11 g fat, 50 g carbohydrate) was consumed to match the caloric deficit between the LCD and LCD + INT interventions. Replacement was based on a previously reported 340 kcal expenditure per 60 min INT session in overweight/obese adults [25].

### 2.4. Metabolic Control

Participants were instructed to refrain from caffeine or alcohol consumption as well as strenuous exercise 48 h prior to testing. Participants were also instructed to refrain from taking any medications or dietary supplements 24 h prior to reporting to the Clinical Research Unit. Participants were instructed to record 3 days of habitual diet before pre-testing. In addition, individuals were instructed to consume approximately 250 g of carbohydrates on the day before pre-testing to minimize influence of muscle glycogen on insulin action. The last training bout was performed approximately 24 h before metabolic testing.

### 2.5. Cardiorespiratory Fitness

VO_2_ peak was determined using an exercise test to volitional exhaustion on a cycle ergometer with indirect calorimetry (Carefusion, Vmax CART, Yorba Linda, CA, USA) [20]. Work output was increased by 25 watts every 2 min until the subject met volitional exhaustion, respiratory exchange rate > 1.1 or cadence < 60 rpm. Heart rate (HR) and blood pressure were obtained at rest and HR was continuously monitored using a 12-lead EKG. 

### 2.6. Body Composition

Following an approximate 4 h fast, body weight was measured to the nearest 0.01 kg on a digital scale with minimal clothing and without shoes. Height was measured with a stadiometer. Body fat and fat-free mass (FFM) was determined using the BodPod (BodPod, Cosmed, CA, USA), and hydration was measured using the InBody 770 Body Composition Analyzer (InBody CO, Cerritos, CA, USA). 

### 2.7. Pancreatic ß-Cell Function

Participants arrived at the Clinical Research Unit after an overnight fast and underwent a 2 h 75 g oral glucose tolerance test (OGTT). Resting metabolic rate was also determined using indirect calorimetry. Blood samples were obtained from an antecubital vein at 0, 30, 60, 90, and 120 min for the determination of glucose, insulin, and C-peptide concentrations. GLP-1_Active_ was measured during 0, 30 and 60 min of the OGTT to assess incretin effects, and free-fatty acids (FFA) were measured at 0 min. Total area under the curve (tAUC) was calculated using the trapezoid method. Skeletal muscle insulin resistance (IR) was estimated using the inverse of the Matsuda Index (IR_Skm_ = 1/Matsuda index) [26]. Hepatic (IR_Hep_) and adipose (IR_Adip_) insulin resistance were also estimated using fasting glucose and fasting FFA multiplied by insulin, respectively [20,27]. Insulin secretion was reconstructed by deconvolution from plasma C-Peptide (Pre-hepatic insulin secretion: ISR) [28]. C-peptide was utilized to characterize insulin secretion to minimize influences of insulin clearance on pancreatic function assessment. Early- and total-phase glucose-stimulated insulin secretion (GSIS) were calculated by dividing the ISR tAUC by glucose tAUC during the first 30 and total 120 min of the OGTT. The early- and total-phase disposition index (DI) were used to characterize ß-cell function, each was calculated relative to skeletal muscle insulin resistance (DI_Skm_ = GSIS/IR_Skm_), hepatic insulin resistance (DI_Hep_ = GSIS/IR_Hep_) and adipose tissue insulin resistance (DI_Adip_ = GSIS/IR_Adip_) as previously described by our group [20].

### 2.8. Biochemical Analyses

All samples were immediately centrifuged for 10 min at 15,000× *g* 4 °C and stored at −80 °C for later analyses. Blood glucose was collected in lithium-heparinized vacutainers and immediately analyzed by a glucose oxidase assay (YSI Instruments 2700, Yellow Springs, OH, USA). Plasma insulin and C-peptide were collected in EDTA tubes with a protease inhibitor aprotinin added and subsequently analyzed by ELISA and chemiluminescence, respectively. Circulating GLP-1_Active_ was also collected in EDTA tubes with dipeptidyl peptidase-4 inhibitor and aprotonin for analyses using ELISA (EMD Millipore, MA, USA). FFA was analyzed using a colorimetric assay (Wako Chemicals, Richmond, VA, USA). To minimize inter-assay variability, pre- and post-intervention samples for each participant were run in duplicate on the same plate. 

### 2.9. Statistical Analysis

Data were analyzed by using SPSS Version 24 (IBM Statistics, Chicago, IL, USA). Twenty-six participants (*n* = 13 LCD, *n* = 13 LCD + INT) completed the 2-week interventions. Two participants were excluded from analyses due to non-compliance (*n* = 1 LCD) and failure to obtained blood post-intervention (*n* = 1 LCD + INT), and three were excluded as outliers from LCD + INT for DI calculations. Comparisons of group baseline variables were performed using independent samples *t-*test. Normality was tested using Q-Q plots and the Shapiro-Wilk test. Non-normally distributed data (all IR and DI data) were log-transformed before analysis. Pre- and post-measures between LCD and LCD + INT were compared using a repeated measures ANOVA. Significant interactions were followed up with tukey post hoc analysis. Bivariate linear regression analysis was used to determine associations. Significance was accepted as *p* < 0.05. Data are presented as mean ± SD.

## 3. Results

### 3.1. Diet and Exercise Compliance

The caloric deficit was not different between interventions (Interaction: *p* = 0.47, Table 1). Percent energy intake from fat decreased, whereas energy from carbohydrates and protein increased in both interventions when compared with pre-intervention diet (Table 1, Time: *p* ≤ 0.01). Participants in the LCD + INT intervention completed all exercise sessions (HRpeak: 82.5 ± 2.2% and rating of perceived exertion (RPE) 12.8 ± 0.7).

### 3.2. Body Composition and Fitness

Both interventions decreased body mass, but this reduction was about 1% greater following the LCD when compared with LCD + INT (Interaction: *p* = 0.02, Table 1). Although neither intervention altered body fat % (Time: *p* = 0.46), FFM increased slightly after LCD + INT when compared with LCD (Interaction: *p* = 0.02, Table 1). RMR did not change pre- to post-intervention (LCD −46.4 ± 339.3, LCD + INT 115.1 ± 255.4; *p* = 0.60). As expected, VO_2_ peak increased about 7% following LCD + INT, with no change after LCD (Interaction: *p* = 0.03). 

### 3.3. Glucose and Hormone Responses

**Figure 1:**
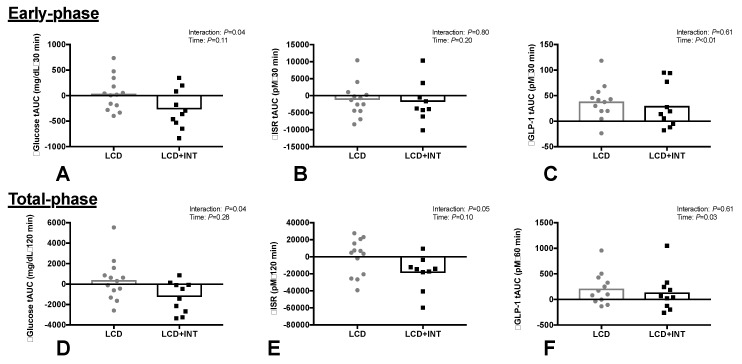
Glucose tolerance tAUC (A,D), insulin secretion rate (ISR: B,E) and GLP-1 tAUC (C,F) for early- and total-phase responses to an OGTT before and after a 2-week period of either a low-calorie diet (LCD) or LCD plus interval training (LCD + INT) in obese adults.

LCD + INT reduced early- and total-phase glucose tAUC by 11% and 6% compared with LCD (Figure 1, Interaction: *p* = 0.04). The % participants with prediabetes based on fasting were (LCD: *n* = 15%; LCD + INT: *n* = 7%) and/or 2 h glucose (LCD: *n* = 8%; LCD + INT: *n* = 23%). Although neither intervention impacted early-phase ISR tAUC (Time: *p* = 0.20), LCD + INT, but not LCD, reduced total-phase ISR tAUC by 15% (Figure 1, Interaction: *p* = 0.05). Both interventions decreased circulating insulin, but not C-peptide, responses to the OGTT (Table 2). LCD and LCD + INT increased GLP-1 tAUC (Figure 1*,* Time: *p* < 0.05). LCD and LCD + INT increased total-phase HC by 12% (Table 2, Time: *p* = 0.01), but not early-phase.

### 3.4. Insulin Resistance

Both LCD + INT and LCD reduced skeletal muscle and liver insulin resistance by approximately 16 ± 28% and 17 ± 31%, respectively (Time: *p* < 0.05, Table 3). Neither intervention, however, affected adipose insulin resistance, although fasting FFA tended to be higher following LCD compared with LCD + INT (LCD: 0.14 ± 0.2 vs. LCD + INT: 0.0 ± 0.1 mEq/L, Interaction: *p* = 0.07).

### 3.5. Pancreatic ß-Cell Function

**Figure 2:**
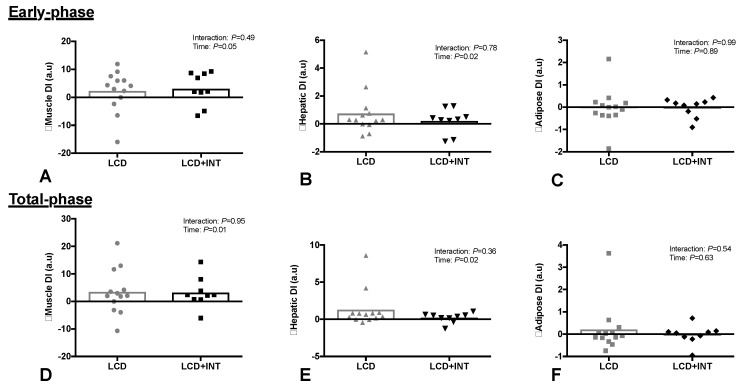
Effect of 2 weeks on a low-calorie diet with (LCD + INT) and without (LCD) interval exercise training on skeletal muscle (**A**,**D**), hepatic (**B**,**E**) and Adipose (**C**,**F**) disposition index (DI). Which was calculated as glucose-stimulated insulin secretion adjusted for skeletal muscle, liver and adipose insulin resistance. Data are changes from baseline for individual (dots) and mean groups (bar).

LCD + INT and LCD interventions did not change early-phase or total-phase GSIS (Table 2). However, LCD + INT and LCD increased early-phase (Time: *p* = 0.05) and total-phase DI_skm_ (Time: *p* = 0.01, Figure 2). Both interventions also increased DI_Hep_ for the early-phase (Time: *p* = 0.02) and total-phase (Time: *p* = 0.02, Figure 2). Adipose tissue DI_Adi_ did not change after either intervention (Figure 2). 

### 3.6. Correlation Analyses

**Figure 3:**
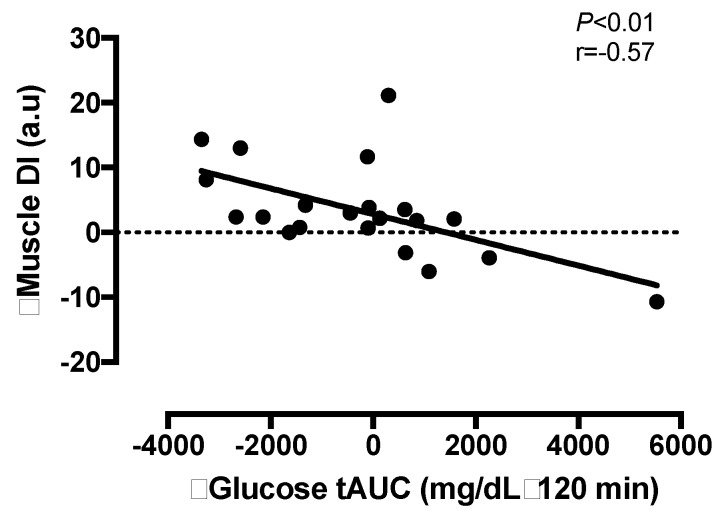
The relationship between the change in total skeletal muscle disposition index (DI) and glucose tolerance (tAUC) after a 2-week period of either a low-calorie diet (LCD) or LCD plus interval training (LCD + INT) in obese adults.

Intervention-induced changes in total DI_Skm_ were not related to changes in energy intake (*r* = 0.07, *p* = 0.85), energy deficit (*r* = 0.00, *p* = 0.93) or to the changes in body mass (*r* = −0.08, *p* = 0.61), VO_2_ peak (*r* = −0.09, *p* = 0.63) or body fat % (*r* = −0.05, *p* = 0.66). Whereas, the changes in FFM tended to be related to total DI_Skm_ (*r* = 0.37, *p* = 0.08). The changes in glucose tolerance (tAUC_120_) were related to the changes in total DI_Skm_ (*r* = −0.57, *p* <0.01, Figure 3). Increased GLP-1 was not related to the changes in total DI_Skm_ (*r* = −0.10, *p* = 0.81). Intervention-induced changes in ISR and GSIS were not related to any study outcome (all *p* > 0.10). 

## 4. Discussion

The major finding of the present study was that 2 weeks of an LCD combined with INT reduced glucose and ISR tAUC responses to an OGTT compared to a LCD alone (Figure 1). However, both interventions significantly improved early- and total-phase GSIS when adjusted to skeletal muscle and hepatic insulin resistance (Figure 2). Improvements in total phase skeletal muscle DI were also related to improvements in glucose tolerance (Figure 3), but not to the changes in GLP-1, body mass or fitness. This suggests that combining INT with a LCD is clinically better for glucose control through, in part, a pancreatic ß-cell function adaptation. These findings are of clinical relevance since the oral disposition index is a strong predictor of future risk for type 2 diabetes [8] and obese individuals may have up to 50% reduced ß-cell function when compared with lean counterparts [29]. While our findings suggest not all individuals improve to the same magnitude following treatment, the improvement seen in the present study suggests that acute energy deficit via LCD is a stimulus for GSIS adjusted to skeletal muscle and hepatic insulin resistance and combining interval exercise may be additive for reducing the rate at which insulin is secreted. Interestingly, interval exercise training improves insulin secretion relative to changes in skeletal muscle and/or hepatic insulin resistance [13,19]. A consideration with these prior studies is that they measured pancreatic function using either the hyperglycemic clamp [19] or fasting/OGTT measures with insulin [13]. Together, these studies potentially limit understanding C-peptide derived measures of pancreatic function under physiologic conditions of feeding. In addition, overall energy balance was not controlled for, thereby raising question of whether exercise or exercise-induced energy deficit, improved insulin secretion [13,18]. Interestingly prior work by Weiss et al. [10] showed greater effects of combining caloric restriction and exercise training (to elicit ~6% weight loss) on insulin sensitivity, but not ß-cell function in overweight individuals. Our findings add to this literature by showing that 2 weeks of either LCD + INT or LCD enhances skeletal muscle and hepatic DI independent of clinically meaningful weight loss, suggesting that early on in response to lifestyle intervention, the pancreas improves favorably.

Weight loss and negative energy balance have independently been suggested to improve glucose regulation in adults with obesity [10,23,30]. While we cannot tease the effect of negative energy balance per se from that of stable weight loss in this intervention, the LCD intervention promoted greater reductions in body mass (albeit by ~1%) when compared to the LCD + INT. Both groups reduced caloric intake comparably between groups, and only the exercise intervention consumed a post-meal of about 350 kcal to equate energy availability. Thus, it would be expected that both groups lose comparable weight. Interestingly, INT preserved and increased FFM compared with LCD alone, suggesting that exercise increased muscle hypertrophy given there was no change in hydration status. While it remains possible that people undergoing INT decreased habitual activity more so than LCD alone, this is unlikely as this group gained about 7% in aerobic fitness. Regardless, of the subtle difference in weight loss, there was no difference in body fat, and this weight loss did not correspond to greater improvements in glucose tolerance or ß-cell function. This indicates that weight loss is not the sole requirement for improved GSIS adjusted for skeletal muscle or hepatic insulin resistance following lifestyle interventions, which is similar to prior work [10]. Thus, our results highlight that when the energy deficit is matched, regardless of whether diet or exercise induced, there are similar improvements in ß-cell function.

Based on previous work, it was hypothesized that caloric restriction and/or exercise may differentially impact tissue-specific indices of insulin resistance and pancreatic ß-cell function [20,31]. Herein we characterized early and total phase GSIS to depict the readily available pool of insulin to be released upon glucose-stimulation compared with the synthesis of new insulin in response to ambient fluctuations in circulating glucose. Interestingly, both interventions improved skeletal muscle and hepatic DI during early and total phase, with no effect of either short-term intervention on adipose DI. The lack of change in adipose tissue DI may be due to the minimal effect of the present short-term interventions on body fat and adipose insulin resistance. The changes in skeletal muscle DI are in agreement with previous lifestyle interventions [13,14,32,33], and reiterates the importance of diet and exercise for improving the ability of the pancreas to secrete insulin relative muscle and liver during the post-prandial period. Weiss et al. [10] observed greater improvements for insulin sensitivity when a LCD was combined with exercise training in obese adults. In contrast, we observed similar improvements for peripheral insulin resistance with LCD + INT and LCD. However, it is difficult to compare these studies as the intervention was much longer (2-week versus 20-week) and involved significant weight loss (6−8%) compared to the present study (< 3%). Moreover, we did note significant reductions in fasting glucose and insulin following LCD + INT and LCD alone. This suggests improvements in hepatic insulin resistance during caloric restriction, and highlights that energy deficit is important for improving hepatic glucose production in obese adults prior to overt hyperglycemia.

Skeletal muscle is considered a primary tissue regulating glucose metabolism. Most glucose that is ingested following a meal is taken up by peripheral tissues such as skeletal muscle [34]. In the present study, improvements in skeletal muscle DI were significantly associated with improved glucose tolerance and tended to be related to increased FFM. Indeed, following LCD + INT there was an 11% decrease in glucose tAUC and 2% increase in FFM. Importantly, this was not related to changes in total body water, which did not change after either intervention. Given the importance of skeletal muscle in the disposal of glucose after a meal [34], our work highlights the effect of INT exercise in preserving FFM during a LCD while concomitantly improving pancreatic function. Previous work has highlighted the feasibility of INT when prescribed at their own relative intensity in at risk populations [13,25]. For example, Karstoft et al. [25] employed a similar INT protocol, but non-supervised, over a 16-week exercise intervention in adults with T2D and found improved glucose regulation. However, this current study is the first to combine INT with caloric restriction. The present study suggests INT and caloric restriction is feasible over 2 weeks in adults with obesity, as all adults successfully completed the intervention with high adherence. Moreover, our results highlight that when exercise is performed relative to their maximal capabilities, individuals are able to perform INT with health benefit. This study was not designed to assess the mechanism by which skeletal muscle could impact insulin secretion, but one possible explanation could relate to a change in the release of myokines [35]. Indeed, several myokines have been identified to facilitate tissue cross talk and promote pancreatic function after exercise training [36]. Whether caloric restriction induces changes in myokines comparably to exercise, with or without caloric restriction, requires further attention in order to gain mechanistic understanding of lifestyle on chronic disease risk.

GLP-1 and GIP are responsible for a large proportion of GSIS, and both are reduced in obesity [37,38]. Previous work [32,39] shows that improvements in GSIS after lifestyle-induced weight loss is linked to alterations in GIP secretion. However, more recent work has raised the question as to whether it is GIP or GLP-1 that plays the predominate role in altering insulin secretion following diet and/or exercise interventions [40]. Given recent data which highlights the role of GLP-1 in mediating changes in insulin secretion independent of weight loss [41], we hypothesized that the changes in pancreatic ß-cell function following our treatments may be mediated by changes in GLP-1. In the present study, active GLP-1 increased ~16% after both interventions, suggesting energy deficit, not exercise, improves GLP-1 concentrations in obese adults. For example, changes in the gut microbiota in response to caloric restriction may explain the observed increases in GLP-1 secretion [42]. The clinical relevance of this is unclear as increased GLP-1 did not correlate with the changes in GSIS or DI. Conversely, Weiss et al. [10] observed a decrease in postprandial GLP-1 after long-term caloric restriction, but not after combined caloric restriction and exercise. The present study suggests that changes in GLP-1 are more likely related to the caloric restriction rather than the addition of exercise. Moreover, our data are consistent with some [43,44] but not all [45] studies that have shown exercise to have little effect on raising gut hormones. Collectively, these data suggest improvements in pancreatic function are independent of GLP-1, and further work is required to understand how LCD and/or INT exercise improve insulin secretion. 

A potential limitation of this study is the small sample size and the predominantly female population, thereby limiting the generalizability of our findings. While the male participant in the present study responded similarly to women, we are underpowered to definitively test sex effects and further work is needed. Within the women studied there were a mixture of pre- and post-menopausal women, with equal numbers in each group. Although groups were relatively matched, it remains possible that LCD versus LCD + INT may induce different effects. Moreover, the effects of menstrual status on ß-cell function within a short-term intervention are unknown, and require further investigation. Another point to consider is that the energy deficits are based on 14 day food logs as we did not directly control feeding for the 2-week interventions nor did we use doubly-labeled water to estimate energy expenditure. Thus, it remains possible that our estimations of the energy deficit are under-/over-estimated. However, we did not see energy intake or weight loss after the intervention relate to glucose tolerance or ß-cell function. Glucose regulation was measured approximately 24 h following the last exercise bout. As such, it is possible that some of the LCD + INT intervention effect is due to the acute residual effects of exercise. However, this would seem unlikely given insulin resistance, GSIS and pancreatic function measures were improved comparably between treatments. The present study used an oral glucose load to physiologically characterize the effects of our interventions on ß-cell function in addition to being dependent on the incretin hormones. Further work using the intravenous glucose tolerance test or hyperglycemic clamp is needed to confirm these results. Moreover, with reductions in circulating insulin being possibly due to increased hepatic clearance, we may have over-estimated the change insulin sensitivity. However, this approach is a validated calculation that is highly correlated with the gold-standard euglycemic-hyperinsulinemic clamp [26].

## 5. Conclusions

In conclusion, we demonstrate for the first time that 2 weeks of combined caloric restriction and INT training improves glucose tolerance more than LCD alone. The reason for such improvements in glucose metabolism may relate to glucose-stimulated insulin secretion when scaled to skeletal muscle, but not liver or adipose. Interestingly, these changes in skeletal muscle DI tended to be related to increases in lean body mass; highlighting the importance of skeletal muscle function for glucose control. However, additional work is required to elucidate the exact mechanism(s) by which LCD + INT and LCD alone improves ß-cell function to optimize diabetes prevention.

## Figures and Tables

**Table 1:** Pre- and post-intervention characteristics for the LCD and LCD + INT groups.

	LCD		LCD + INT		Time (*p*-Value)	Interaction (*p*-Value)
	Pre	Change	Pre	Change		
N, (M/F)	12 (1/11)	11 (0/11)				
Age (year)	45 ± 12	47 ± 14				
**Body Composition**						
Height (cm)	166 ± 6	168 ± 6				
Body mass (kg)	103.2 ± 15.8	*−2*.9 ± 0.9	107.3 ± 20.2	−1.7 ± 1.3	<0.01	0.02
BMI (kg/m^2^)	37.4 ± 6.3	*−1*.0 ± 0.3	38.0 ± 7.7	−0.3 ± 0.8	<0.01	<0.01
Body fat (%)	46.7 ± 6.4	−0.3 ± 0.9	48.2 ± 5.7	0.1 ± 1.1	0.46	0.20
Body fat (kg)	49.0 ± 12.7	−1.4 ± 1.0	52.2 ± 14.6	−0.6 ± 1.0	<0.01	0.07
Fat-free mass (kg)	51.4 ± 6.3	−0.7 ± 0.4	52.3 ± 9.0	1.0 ± 0.9	0.05	0.02
Body Water (L)	41.3 ± 5.7	−0.8 ± 0.5	40.0 ± 5.8	−0.7 ± 1.0	<0.01	0.67
**Fitness**						
VO_2_ peak (L/min)	1.9 ± 0.5	−0.1 ± 0.3	1.9 ± 0.4	0.1 ± 0.3	0.98	0.04
VO_2_ peak (mg/kg/min)	19.7 ± 4.9	−0.5 ± 1.6	18.6 ± 4.9	1.4 ± 2.1	0.28	0.03
**Bloods**						
FPG (mg/dL)	96.6 ± 4.8	−3.6 ± 8.0	97.7 ± 7.7	−2.2 ± 6.7	0.01	0.93
2 h PG (mg/dL)	110.5 ± 21.7	7.7 ± 22.7	123.2 ± 21.3	−5.1 ± 24.5	0.61	0.13
FIns (µU/mL)	15.4 ± 9.0	−2.9 ± 6.0	21.7 ± 20.1	−4.1 ± 7.7	0.05	0.73
2 h Ins (µU/mL)	76.1 ± 52.0	−0.9 ± 22.7	148.0 ± 110.4	−26.3 ± 61.1	0.15	0.17
FC-pep (ng/mL)	2.2 ± 0.7	−0.3 ± 0.5	2.6 ± 1.3	−0.4 ± 0.5	<0.01	0.84
2 h C-pep (ng/mL)	8.0 ± 2.9	0.5 ± 1.9	11.8 ± 4.0	−2.5 ± 2.5	0.04	<0.01
**Diet**						
Energy intake (kcal)	2243 ± 759	−854 ± 768	2110 ± 648	−639 ± 580	<0.01	0.47
CHO (%)	46 ± 7	7 ± 7	46 ± 11	7 ± 10	<0.01	0.89
Protein (%)	16 ± 4	2 ± 4	16 ± 4	3 ± 1	0.01	0.61
Fat (%)	38 ± 7	−9 ± 7	39 ± 8	−12 ± 2	<0.01	0.37

Low-calorie diet (LCD), LCD plus interval training (LCD + INT), BMI = Body mass index, VO_2_ peak = Peak oxygen uptake, FPG = Fasting plasma glucose, FPI = Fasting plasma insulin. CHO = carbohydrate.

**Table 2:** Early-phase and total-phase responses to an OGTT, before and after each 2-week intervention.

	LCD		LCD + INT		Time (*p-*Value)	Interaction (*p-*Value)
	Pre	Change	Pre	Change		
**Early-Phase Responses**					
Insulin tAUC_30_ (µU/mL•30 min)	2206 ± 1154	−408 ± 885	2544 ± 1238	−368 ± 670	0.07	0.66
C-peptide tAUC_30_ (ng/mL•30 min)	148 ± 46	−11 ± 35	164 ± 57	−22 ± 35	0.25	0.62
ISR (pM •30 min)	20652 ± 1878	−1211 ± 4828	23647 ± 2299	−1804 ± 5938	0.20	0.80
GSIS (pM•min/mg/dL)	5.31 ± 1.29	−0.26 ± 1.01	6.50 ± 1.41	−0.05 ± 1.52	0.44	0.55
HC (µU/mL•mg/dL^−1^)	12.5 ± 5.1	−0.6 ± 3.3	13.9 ± 3.7	−0.4 ± 2.0	0.44	0.91
**Total phase responses**					
Insulin tAUC_120_ (µU/mL•120 min)	11094 ± 5665	−1685 ± 3882	14872 ± 8951	−2525 ± 3185	0.01	0.71
C-peptide tAUC_120_ (ng/mL•120 min)	895 ± 260	−10 ± 167	1092 ± 340	−109 ± 122	0.14	0.22
ISR (pM•120 min)	96013 ± 8088	−283 ± 2129	130861 ± 14511	−18956 ± 20239	0.10	0.05
GSIS (pM•min/mg/dL)	5.94 ± 1.73	0.23 ± 0.91	7.95 ± 1.68	−0.52 ± 1.1	0.24	0.41
HC (µU/mL•mg/dL^−1^)	11.7 ± 3.8	−1.7 ± 2.7	13.4 ± 4.5	−1.2 ± 2.2	0.01	0.60

Low-calorie diet (LCD), LCD plus interval training (LCD + INT), tAUC = total area under the curve, ISR = Insulin Secretion Rate, GSIS = glucose-stimulated insulin secretion, HC = Hepatic Clearance.

**Table 3:** Insulin resistance before and after each 2-week intervention.

	LCD		LCD + INT		Time (*p*-Value)	Interaction (*p*-Value)
	Pre	Change	Pre	Change		
Skeletal muscle IR	0.38 ± 0.21	−0.08 ± 0.13	0.64 ± 0.53	−0.16 ± 0.19	<0.01	0.24
Hepatic IR	3.3 ± 2.1	−1.0 ± 1.7	5.8 ± 5.7	−1.3 ± 2.5	0.01	0.59
Adipose IR	7.6 ± 4.6	0.1 ± 2.9	13.3 ± 13.3	−2.4 ± 6.2	0.14	0.13

Low-calorie diet (LCD), LCD plus interval training (LCD + INT), IR = Insulin Resistance.
